# Electronic Cigarette Burns: A Case Report and Review of Current Literature

**DOI:** 10.1155/2019/4231764

**Published:** 2019-10-21

**Authors:** Rachel Michael, Nabil Ebraheim, Jacob Maier, Mina Tanios, Anthony Kouri

**Affiliations:** University of Toledo Medical Center Department of Orthopaedic Surgery, 3000 Arlington Ave Toledo, OH 43614, USA

## Abstract

Due to the development of electronic cigarettes and their use in our patient population, this article seeks to evaluate the safety and associated morbidity that may result from their use. This article also presents a patient case regarding an explosion of an electronic cigarette battery and the resultant injury and deformity that occurred.

## 1. Introduction

In recent years, Electronic Nicotine Delivery Systems (ENDS), also known as electronic cigarettes (e-cigarettes) have gained increased popularity. While viewed as a healthier alternative to traditional tobacco cigarettes, ENDS present new dangers. In 2016, the National Electronic Injury Surveillance System (NEISS) reported 26 cases of ENDS-related burn injuries that were presented to emergency departments, which translates to a national average of 1007 ENDS-related burn injuries [1]. Corey et al. also report that the majority of these burns (77.3%) were located on the patient's upper trunk/lower leg, which is a result of explosions occurring in the shirt or pant pockets. Numerous case studies have shown that, in addition to the thigh, the genitalia are often injured in these types of explosions [2, 3]. Injuries to the hands and face are also common when the ENDS explodes during use [4, 5]. In addition to burns, maxillofacial fractures have also been documented [6]. Researchers attribute the cause of these explosions to the lithium ion battery that powers the device [7]. This mechanism contributes to the mixed thermal and alkali chemical burns seen in patients [8]. This article focuses on the morbidity-associated burns from ENDS and does not take into account the overall prevalence of burn injuries.

Nicotine-free e-cigarettes appear to be more popular than their nicotine counterparts among the younger generation. One study showed that out of all teenagers that reported using e-cigarettes, 72% used products without nicotine (versus 28% who used products with nicotine) [9]. Ferrari et al. found that there were no immediate adverse effects from nicotine-free e-cigarettes, with respect to fractional concentration of exhaled carbon monoxide and nitric oxide [10]. The use of e-cigarettes has also been shown to mimic cancer. Ring Madsen et al. presented a case that suggests e-cigarettes can induce an inflammatory response similar to responses found in metastatic cancer [11]. Similarly, Fracol et al. found that e-cigarette vapor was cytotoxic to endothelial cells independent of nicotine content [12].

## 2. Case

**Figure 1:**
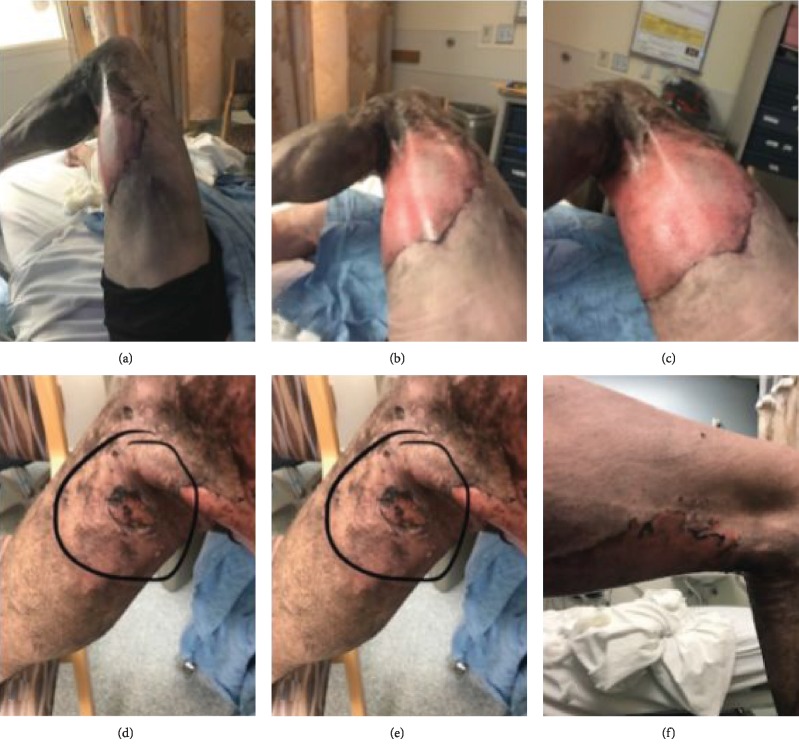
Images (a–f) are the presenting burn injuries on the left lower extremity.

**Figure 2:**
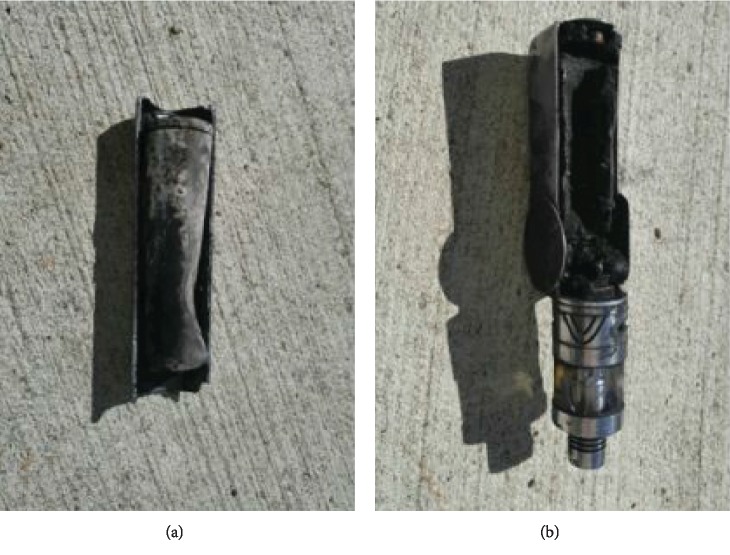
Images (a, b) represent the electronic cigarette after it exploded.

This case was chosen due to its unique historical presentation and resultant morbidity. A 40-year-old male presented to the emergency department with severe burns on the left posterior thigh that resulted from the spontaneous combustion of an ENDS in his left front pant pocket (Figures 1 and 2). The patient states that the device was turned off while in his pocket. The patient was a nonnicotine e-cigarette user. Due to the nature of his injury, the patient was transported to a nearby hospital burn unit. His injuries were treated with multiple debridements both bedside and in the operating theatre. The patient then underwent a split thickness autograft and additional use of an allograft matrix four days after injury. Following the procedure, the left thigh graft remained viable and had been incorporated fully; however, there was some evidence of graft hypertrophy and signs of contracture around the boundary of the graft.

One month after injury, the patient continued to have intermittent pain, irritation, and numbness over the left posterior thigh burn site and has not returned to a normal gait. The patient states that since his injury, he has noticed a drastic decline in his activities and ability to exercise at the level he did prior to his injury. He also has cosmetic concerns about his burn scars and contractures and thus wears clothes that cover the exposed sites.

**Figure 3:**
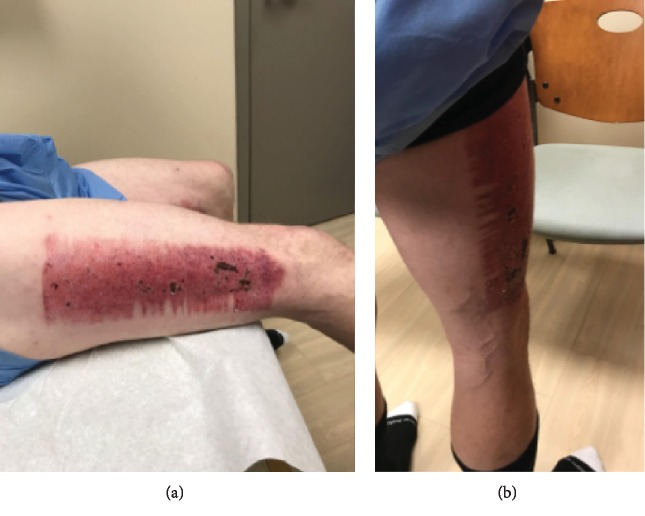
The right thigh site of the split thickness skin graft donor site. The patient is one month after grafting.

**Figure 4:**
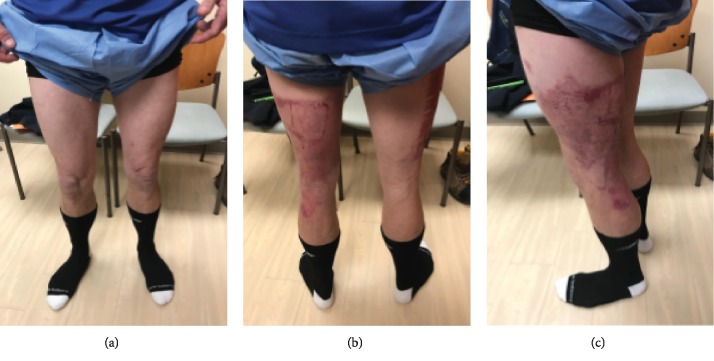
Anterior, posterior, and lateral gross images of the patient bilateral lower extremities one month after injury. The discrepancy in quadriceps volume can be seen in (a). (b) demonstrates the residual scar from his burns and grafting on the left posterior thigh. (c) is the lateral view of the left lower extremity injury after grafting.

Patient examination one month after his injury demonstrated a mildly antalgic gait with external rotation of the left lower extremity with external foot progression compared to the contralateral side. The patient had full tone and motor strength throughout the bilateral lower extremities. There was no cyanosis or edema noted on his examination. Focused examination of the right lower extremity demonstrated a scab over the skin graft harvest site on the anterior thigh (Figure 3). At the donor site on the right thigh, the patient experienced some pain which manifests as tenderness and redness. He was neurovascularly intact distally. Of note, the right lower extremity had quadriceps atrophy compared to the contralateral side (Figure 4).

**Figure 5:**
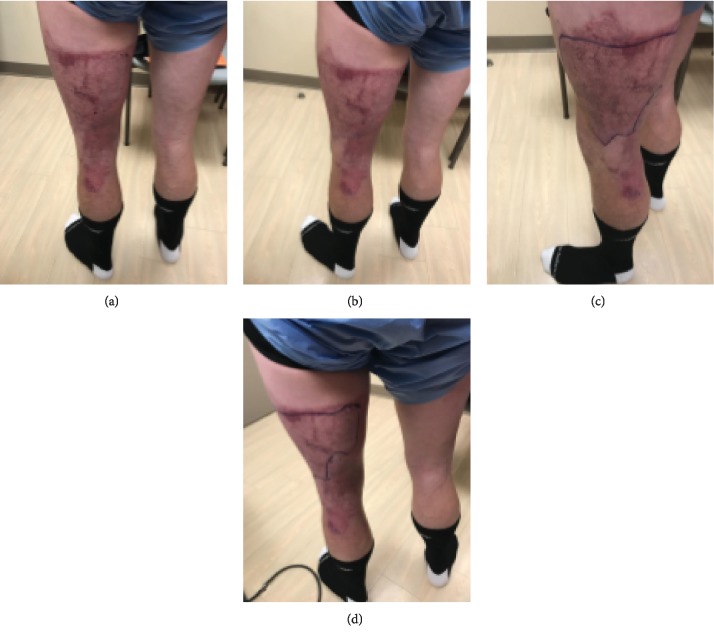
Views of the affected left lower extremity one month after injury. (c, d) show the outline of the graft recipient site.

The left lower extremity demonstrated healing at the skin graft recipient site with mild contraction over the posterior knee and thigh. The graft spanned the posterior aspect of the knee joint and contributed to the loss of terminal extension of the knee joint. There was no surrounding erythema. On the left lower extremity, the quadriceps muscle appears larger due to fascial defects (Figures 4 and 5). Clinically, there was also evidence of iliotibial band tightness compared to the contralateral extremity which may also contribute to the lack of full mobility. Knee range of motion was assessed and was 15-110 degrees on the left and 5-130 degrees on the right (Table 1). The patient had bilateral hip extension to neutral.

If his symptoms do not respond to conservative measures with physical therapy and scar massage, the patient may require surgical interventions such as revision grafting, iliotibial band release, or possible tendon and skin z-plasties.

The patient also indicated that he is having difficulty coping with his injury. The cosmetic appearance of his graft and donor site is of great emotional concern to the patient. He was recommended for psychological evaluation and treatment to help him cope with the mental trauma associated with his injury.

## 3. Discussion

Cases of severe burns from spontaneous combustion of ENDS are becoming a concern with their increasing popularity as alternatives to traditional cigarette smoking. A large percentage of these explosions occur in the pants or shirt pockets which makes the severe burns on the thigh and chest or upper extremity a common presentation. The scar contractures around the knee and hip joints pose a significant functional limitation. The psychiatric effects of the incurred trauma and resultant cosmetic deformity are not well documented in the current literature but present a significant issue for these patients. Our patient struggled with the appearance of burns on his legs, yet many patients have been documented to have prominent burns on their faces and hands. The physiological trauma of this injury cannot be overlooked. It is also postulated that less severe burns may occur with more frequency and are not included in the studies of severe burns requiring surgical treatment. These less severe burns may be treated by family practice or emergency department personnel and are thus not included in the present study population. Further investigation is needed on all burns from ENDS.

It is our intention with this article to demonstrate the dangers of these devices so that the public and our patients may be better informed. The danger of serious burns is related to the combustibility of the lithium battery that powers the devices, which is unique compared to heat burns from traditional tobacco cigarettes. Thus, there needs to be further investigation to compare chemical burns associated with electronic cigarettes versus traditional cigarette burns. We currently recommend against the use of all ENDS, both nicotine-containing and nicotine-free devices, due to the cytotoxicity of the vapors and the potential for device explosion. However, we understand that these devices will continue to be used within our patient population, and thus, it is our recommendation that guidelines for proper storage and care of the devices should be made.

## Figures and Tables

**Table 1:** Lower extremity range of motion one month after injury.

Lower extremity range of motion		
	Right lower extremity (autograft harvest limb)	Left lower extremity (injured/graft recipient limb)
Hip extension	0°	0°
Hip flexion	110°	110°
Knee extension	5°	15°
Knee flexion	130°	110°
